# Supplementary material for: MetaRibo-Seq measures translation in microbiomes
Source: Nat Commun. 2020 Jun 29;11:3268. doi: 10.1038/s41467-020-17081-z (PMC7324362; doi:10.1038/s41467-020-17081-z)
Supplement: Supplementary file 10 — Supplementary Data 7 [file 41467_2020_17081_MOESM10_ESM.zip › File2/Confidence_High_Taxonomy/132501_out.krona.html]

Javascript must be enabled to view this page.

members
magnitude
magnitudeUnassigned
count
unassigned
taxon
rank

132501\_out

29

29
superkingdom
2

1239
phylum
29

29
186801
class

29
order
186802

29
family
541000

genus
1263
29

species
46228
29

SRS011239\_contig\_number\_contig-100\_1654.174480SRS012273\_contig\_number\_24290SRS013098\_contig\_number\_contig-100\_4126.274063SRS014235\_contig\_number\_10663SRS015663\_contig\_number\_29830SRS015854\_contig\_number\_contig-100\_3777.93073SRS015890\_contig\_number\_25706SRS017916\_contig\_number\_14442SRS018984\_contig\_number\_7242SRS019601\_contig\_number\_16047SRS019685\_contig\_number\_contig-100\_747.222045SRS021948\_contig\_number\_contig-100\_30297.30297SRS022071\_contig\_number\_contig-100\_10790.10790SRS024492\_contig\_number\_contig-100\_703.110631SRS048060\_contig\_number\_3381SRS050752\_contig\_number\_30049SRS051031\_contig\_number\_34527SRS051610\_contig\_number\_3158SRS051882\_contig\_number\_contig-100\_9611.116812SRS062701\_contig\_number\_5750SRS098571\_contig\_number\_contig-100\_8453.187887SRS104197\_contig\_number\_contig-100\_1147.7306SRS142542\_contig\_number\_24630SRS143181\_contig\_number\_1934SRS146764\_contig\_number\_20495SRS147039\_contig\_number\_contig-100\_7043.7044SRS147766\_contig\_number\_9753SRS893172\_contig\_number\_contig-100\_20990.57577SRS971275\_contig\_number\_39487
